# Dynamic Multiscale Boundary Conditions for 4D CT of Healthy and Emphysematous Rats

**DOI:** 10.1371/journal.pone.0065874

**Published:** 2013-06-14

**Authors:** Richard E. Jacob, James P. Carson, Mathew Thomas, Daniel R. Einstein

**Affiliations:** Biological Sciences Division, Pacific Northwest National Laboratory, Richland, Washington, United States of America; University of Giessen Lung Center, Germany

## Abstract

Changes in the shape of the lung during breathing determine the movement of airways and alveoli, and thus impact airflow dynamics. Modeling airflow dynamics in health and disease is a key goal for predictive multiscale models of respiration. Past efforts to model changes in lung shape during breathing have measured shape at multiple breath-holds. However, breath-holds do not capture hysteretic differences between inspiration and expiration resulting from the additional energy required for inspiration. Alternatively, imaging dynamically – without breath-holds – allows measurement of hysteretic differences. In this study, we acquire multiple micro-CT images per breath (4DCT) in live rats, and from these images we develop, for the first time, dynamic volume maps. These maps show changes in local volume across the entire lung throughout the breathing cycle and accurately predict the global pressure-volume (PV) hysteresis. Male Sprague-Dawley rats were given either a full- or partial-lung dose of elastase or saline as a control. After three weeks, 4DCT images of the mechanically ventilated rats under anesthesia were acquired dynamically over the breathing cycle (11 time points, ≤100 ms temporal resolution, 8 cmH_2_O peak pressure). Non-rigid image registration was applied to determine the deformation gradient – a numerical description of changes to lung shape – at each time point. The registration accuracy was evaluated by landmark identification. Of 67 landmarks, one was determined misregistered by all three observers, and 11 were determined misregistered by two observers. Volume change maps were calculated on a voxel-by-voxel basis at all time points using both the Jacobian of the deformation gradient and the inhaled air fraction. The calculated lung PV hysteresis agrees with pressure-volume curves measured by the ventilator. Volume maps in diseased rats show increased compliance and ventilation heterogeneity. Future predictive multiscale models of rodent respiration may leverage such volume maps as boundary conditions.

## Introduction

There is increasing interest in computational fluid dynamics (CFD) modeling of airflow and tissue mechanics in the respiratory tract for purposes of predicting particulate deposition and clearance, uptake of vapors, and disease onset and progression [1]–[5]. Such models can be used to further our understanding of health risks from environmental exposures or alterations in inhaled pharmaceutical uptake caused by disease [6]. Technology is currently available for developing steady-state CFD models of inhalation toxicology in the mammalian respiratory system, including imaging pulmonary architecture, automating image segmentation, and generating surface and volume meshes [5], [7]–[13]. However, a key component, and a major hurdle, to the development of realistic transient CFD models is a quantitative understanding of lung architecture and tissue mechanics, including strain and compliance, and how the parenchyma and airways move during the hysteretic breathing cycle. As transient CFD models are developed, the dynamics and hysteresis of the full breathing cycle must be accounted for with real time-dependent structural data obtained from *in vivo* imaging during breathing. In this way, the different dynamics of inhale and of exhale, which are lost during breath-hold, can be properly incorporated. This paper describes acquisition of dynamic 4D CT images (multi-time-point 3D images acquired without breath-hold), the non-rigid registration of the images, and the calculation of tissue dynamics and local ventilation with high spatial and temporal resolution.

Several different *in vivo* imaging approaches to directly visualize lung motion have been published. For example, proton and ^3^He magnetic resonance imaging (MRI) grid tagging have been used to visualize deformation of the moving lung [14], [15]. MRI has also been used to generate 3D maps of lung motion with the benefits of no ionizing radiation or contrast agents [16]. In general, the MRI approaches have relatively low spatial and/or temporal resolution, and images are typically 2D [17]. Phase-contrast x-ray imaging using monochromatic x-rays is an emerging technique used to directly measure the velocity of lung tissue during the breathing cycle in rodents [18]. This method provides high-resolution velocity maps from 2D projections of the lung at near video-rate temporal resolution. Alternatively, ventilation maps, or volume maps, can be created by use of an inhaled contrast agent, such as oxygen or hyperpolarized gas in MRI [17], [19], [20] or Xe in CT [21], [22] with images typically acquired during breath-hold.

4D CT can be used to measure dynamic local physiological properties such as strain, compliance, inhaled volume, or ventilation (inhaled volume per unit time) [17], [23], [24]. Such an approach provides detailed maps of gas distribution in the lung. Importantly, these maps can be used to indicate regions with irregularities that may be indicative of disease [18]. For example, CT densitometry has been used to calculate local lung compliance in 3D from breath-hold images in irradiated rodents [25].

Lung parenchyma is one of the most viscoelastic biologic materials known in nature. The three predominant mechanisms responsible for this are believed to be the surfactant film, alveolar recruitment, and the extracellular matrix of collagen and elastin [26]–[30]. The role of surfactant was demonstrated by Hildebrandt [31] in air- and saline-filled lungs. The saline treatment showed that lung surfactant plays a major role in lung hysteresis. Without surfactant, collagen and elastin, the primary components in the extracellular matrix [28], are responsible for the relatively small hysteresis in the parenchyma and therefore contribute relatively little to the viscoelasticity of the lung. Alveolar recruitment is not a typical viscoelastic mechanism; nevertheless, an examination of the work of Namati et al. [30] demonstrates that the recruitment process is hysteretic, since the distributions in alveolar size are different at the same pressure during inflation and deflation.

We show from CT-derived volume maps of dynamic breathing (i.e. no breath-holds) in rats that we are able to match the global pressure-volume (PV) behavior over the full breathing cycle, which includes the hysteretic losses due to all three important viscoelastic mechanisms. Previous work has addressed only measurement of static lung compliance, with images acquired during breath-holds [24], during which the lung tissue relaxes and dynamic viscoelastic properties are not manifest. Herein, we image both inhalation and exhalation during dynamic breathing. This difference is significant because hysteretic differences between inhalation and exhalation are lost when the lung is allowed to relax during breath-hold. Furthermore, the mechanisms that contribute to the viscoelasticity can be affected by disease, as we show by altered PV curves in emphysematous rat lungs.

In this paper, we demonstrate a method for preparing rat 4D CT images for non-rigid image-to-image spatial registration (i.e. image warping), we evaluate the quality of the image warp between the two extremes in the breathing cycle, we describe our computation of local volume change beginning with the computation of volumetric strain, and we show the first inhaled air volume maps calculated in healthy and diseased rats using this method. Importantly, we demonstrate for the first time that volume change maps can accurately predict measured lung hysteresis.

## Methods

**Figure 1:**
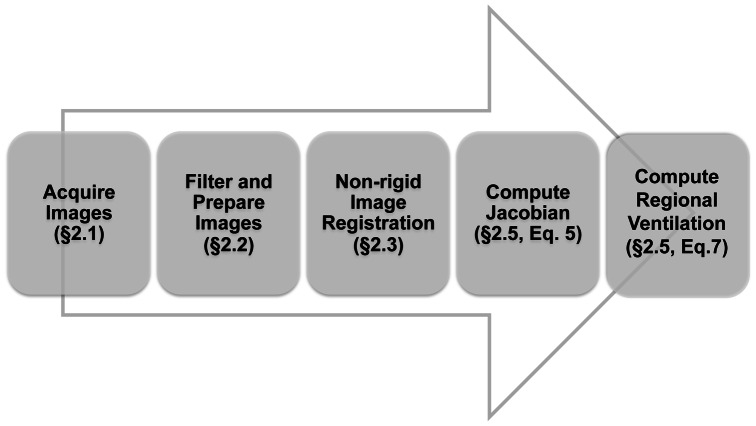
Block diagram illustrating the data processing flow.

Figure 1 shows a block diagram of the data processing flow, with references to section and equation numbers.

### 2.1 Animal Preparation and Imaging

Animal use followed a protocol (2010–23) approved for this study by the Institutional Animal Care and Use Committee of Pacific Northwest National Laboratory. Nine male Sprague-Dawley rats weighing 212±11 g were used. Three rats received an intratracheal dose of 250 U/kg of elastase (EMD Chemicals, Inc., Cat# 324682) in 200 µL saline to the whole lung, three received 50 U/kg of elastase in 200 µL saline to a single lobe, and three received 200 µL saline to the whole lung as a control. Single lobe dosing was monitored using magnetic resonance imaging to verify the dose location. Dose levels were based on previous work in which ^3^He diffusion MRI showed a significant disease response [32].

Three weeks after dosing, rats were imaged with 4D CT. At the time of CT imaging, rats weighed 357±10 g. Details of animal preparation, ventilation, and image collection are described in [33]. In brief, anesthetized and intubated rats were mechanically ventilated using a customized ventilator (CWE Inc. model 830/AP; Ardmore, PA) with air (30% O_2_, 70% N_2_) and 3–4% isoflurane at 60 breaths per minute, with 400 ms inhale and 600 ms exhale durations. Periodic sigh breaths to ∼25 cmH_2_O were delivered to maintain lung recruitment. 4D CT Images were collected at 11 time points throughout the breathing cycle, including at full exhalation and peak inhalation (∼0 cmH_2_O and ∼8 cmH_2_O), without breath-holds. Images were acquired on a GE eXplore 120 scanner with the following settings: 100 kV peak voltage, 50 mA tube current, 16 ms exposure time, and 360 projections with 1 degree angular separation. Imaging time was about 90 minutes. Total radiation dose from imaging was estimated to be 3.3 Gy based on information provided by the vendor. Images were reconstructed to 150 µm isotropic resolution on the GE console using supplied software. Immediately after imaging, rats were sacrificed, and the lungs were cast so that detailed airway architecture could be obtained for CFD modeling efforts.

As described in [33], ventilator volumes were initially acquired during the inhalation cycle only. After the acquisition of these data, the ventilator was upgraded to measure volumes over the full breathing cycle. Six additional untreated rats (one male, five female Sprague-Dawley) were then imaged for purposes of obtaining full ventilator hysteresis data.

### 2.2 Image Preparation for Warping

A direct non-rigid registration, or warp, between image sets is inadvisable because intensity differences develop over the breathing cycle and because differential motion between the lung and the ribcage would have the effect of constraining the warp near the parenchymal boundary [34], [35]. Similar relative motion exists between the parenchyma and the heart. Others have modified their error metric for registration to account for intensity differences [36]. However, this alone does not account for differential motion between the lung and the ribcage nor between the lung and the heart. Furthermore, CFD modeling of airflow and lung function does not require information about the surrounding tissue, which may therefore be masked.

**Figure 2:**
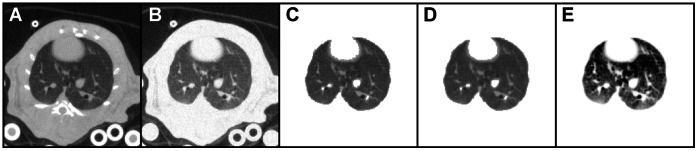
Examples of image processing for registration testing. A) Original image. B) Image with bones removed. C) Image with all background masked. D) Same as C, with Gaussian filter applied prior to masking. E) Same as D, with contrast enhancement applied.

We sought to determine the amount of image processing that was necessary to optimize the warping results by utilizing landmark identification to evaluate warping performance. Five different levels of processing were tried (see Figure 2): A) original images with no processing; B) bone signal replaced with typical surrounding tissue signal, including noise; C) all non-lung background removed, based on a tissue threshold level; D) same as C but with a Gaussian filter of radius = 1 applied; E) same as D but with contrast enhancement using histogram equalization applied. Our results show that this final level of processing (Figure 2E) resulted in the most accurate image registrations.

The specific steps adopted to process the images for warping, such as shown in Figure 2E, were as follows. First, a Gaussian filter (radius = 1) was applied to all the image slices. Next, using a seed point inside the lung, a 3D lung mask was created using the 3D Toolkit plug-in for ImageJ [37]. Then the Region Dilate and Region Erode functions (also in the 3D Toolkit) were applied in succession to fill missed voxels and to smooth the boundaries. Next, the mask was added to the original image using ImageJ’s Image Calculator, effectively saturating all regions of the image outside the lungs while leaving the lungs unaffected (see Figure 2D). Finally, the Enhance Contrast function was applied (with the “equalize histogram” option selected) in order to improve contrast between lung tissue and features in the lung, such as airways and vasculature. This also helped normalize the contrast between source and target images to provide more consistent image feature intensity for registration. This achieves a result similar to the mass preserving error metric presented in [36]. This process was automated with an ImageJ macro and executed in ∼2 minutes on a Mac Pro model 3.1 for an entire 4D image set.

### 2.3 Image Registration

Non-rigid registration, or warping, was performed on each 4D CT image set. The lowest inflation image, acquired at the beginning of the ventilation cycle, was used as the “target” image. Images at the 10 inflation time points were the “source” images. Briefly, we solve a nonlinear deformable registration problem between the target and source images, with mean squared error as the energy. The 3D warping was performed using Plastimatch (www.plastimatch.org), an open source software package for deformable image registration [38]. Optimal warping parameters were determined empirically through dozens of iterations on multiple rat image sets. Final warping parameters were: a first stage with an affine registration with 4×4×4 subsampling and 30 iterations; a second stage with a b-spline registration with 30 iterations, no subsampling, a regularization coefficient of 0.001, and a vector field grid spacing of 10×10×10 pixels. Registration of each 4D image (10 registrations in total) set took ∼45 minutes on a Mac Pro model 3.1.

### 2.4 Registration Accuracy Analysis

To determine the accuracy of the warping, we evaluated the warp results of the highest inflation image, which had the greatest deformation from the target image. Registration accuracy was qualitatively evaluated with relative ease by observing animations (using ImageJ Hyperstacks) of the target and warped images – any motion in the animation was due to registration errors. However, landmark identification was used to quantify registration accuracy in control rats to verify the observed results. One of us identified landmarks throughout the target images of the three control rats (67 total landmarks). The warp quality was then assessed in each of the five cases shown in Figure 2 using one image set. After verifying the best image processing method, the quality of the final registration was independently evaluated in all control rats by three of the coauthors, also through landmark identification. The distances between landmark locations in the target and deformed images, or target registration errors (TRE), were averaged for each landmark. In addition, to give a sense of observer “mouse click” error – the typical error an observer makes when attempting to select a specific landmark with a mouse and cursor – each observer identified landmarks in the identical (unwarped) target image but after a Gaussian filter of radius = 1 had been applied to simulate the blurring effects of warping.

### 2.5 Inhaled Volume Map Calculation

To compute the voxel-by-voxel volume change, we combined computations from two sets of time-series data. The first is the masked and intensity balanced images whose processing is described in Section 2.2. From these images, we computed a kinematic volumetric deformation that was derived from the motion of structures and was therefore independent of Hounsfield (HU) values. The second is the unprocessed series of images that preserves the original HU values and inherently contains an approximation of the percentage of air within each voxel [39]. A full discussion of the rationale behind the interpretation of HU values in CT images of the lung and their role in computing local ventilation can be found in [40]. All computations were performed in BioGeom (https://simtk.org/home/biogeom) on a Mac Pro model 3.1 using MATLAB 2011a.

**Figure 3:**
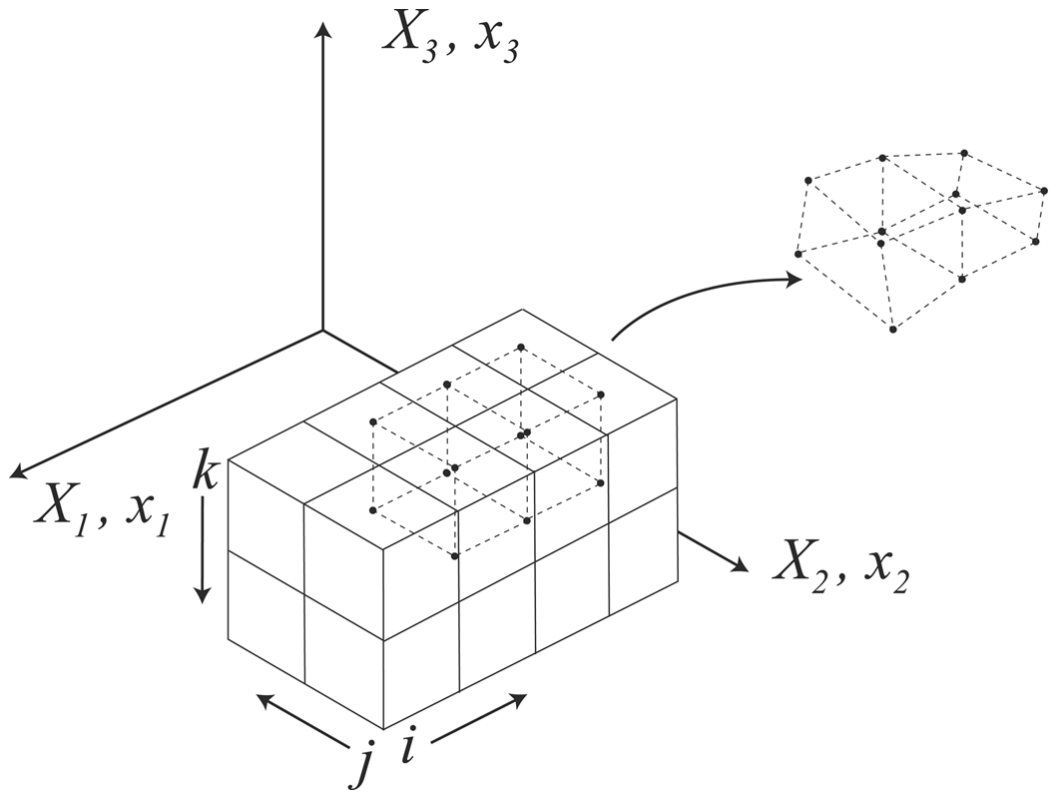
An example of finite element discretization of an image.



Based on the sequence of computed deformation fields described above, we compute the non-linear strain with a finite element discretization [41]. The discretization is set up in the undeformed configuration using isoparametric 8-noded hexahedral elements, defined from the voxel centroids (see Figure 3). Material particles at the voxel centroids, **X** *_a_*, define the initial position of the element nodes as,
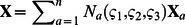
(1)where *N_a_* are standard isoparametric shape functions (with coordinates ς) and *n* is the number of nodes - or in this case voxel centroids. The combination of rigid-body motion and deformation are fully described by the current nodal positions **x** *_a_(t)*

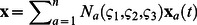
(2)The deformation gradient **F**, which is the fundamental kinematic quantity for finite deformation, is given as:
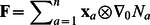
(3)


where 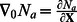 is related to 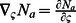 by the chain rule:
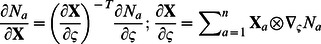
(4)


Given the definition of the deformation gradient (Equation 3), volume change is defined as.
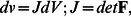
(5)where *J* is the Jacobian. If we reason that all measured volume change in the parenchyma is due to air entering (or leaving) the region, the regional ventilation *rV* can be estimated as



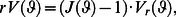
(6)where 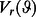 is the voxel volume in the original (*t* = 0 ms) configuration. However, we note that estimation of regional volume change from Equation 6 can be misleading, particularly in the presence of pathology. For example, in emphysema a region of the lung may deform and yet may remain unventilated due to airway collapse. We thus refine the computation of local ventilation by taking into account intensity changes in the original images, which reveal shifts in air and tissue.

Following the approach of [40], regional ventilation is jointly estimated from Equation 5 and from the changes in local air fraction as estimated from the intensity values in the unprocessed images:
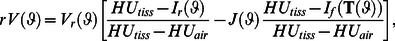
(7)where *HU_tiss_* and *HU_air_* are representative Hounsfield values for air and tissue; *I_r_* and *I_f_* are the intensity values of the voxel φ in the reference (*t* = 0) and the floating (*t* = t) images; and **T**(φ) is the transformation represented by the non-linear warp.

We note that *HU_air_* in this context is the HU value of air in the lung, and not necessarily the defined value of −1000 HU. *HU_air_* values in rat lungs, even in the deep lung and largest airways, rarely reach −1000 HU, likely due to common imaging artifacts such as shading, view aliasing, and beam hardening [42] that can be pronounced in the close proximities of the rat lung. Therefore, the *HU_air_* and *HU_tissue_* values used in Equation 7 were determined for each dose group. A histogram was constructed from the combined images at the highest inflation level, and the mean *HU_tissue_* value was determined from the tissue peak. *HU_air_* was empirically determined as the lowest typically expected value of HU in the group, which value was defined as being two standard deviations below the mean of the isolated lung peak (when fit to a Gaussian curve). For the control, full-lung dose, and single-lobe dose groups, HU_air_ was found to be −781 HU, −700 HU, and −832 HU, respectively, and HU_tissue_ was found to be −122 HU, −126 HU, and −123 HU, respectively.

## Results

**Figure 4:**
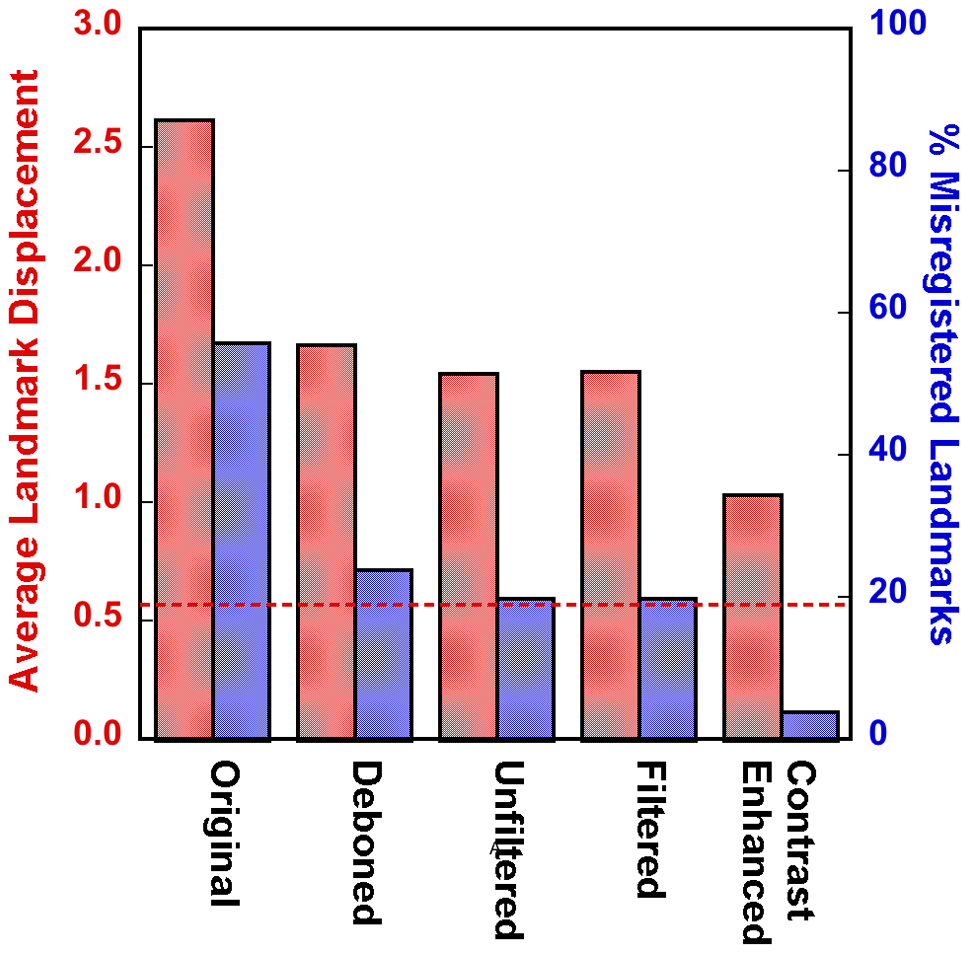
Average landmark displacement (in pixels, red) and the percentage of misregistered landmarks (blue) for the five cases shown in Figure 1. The red dashed line indicates the observer’s average “click error”.

Figure 4 shows the landmark registration results for the five image preparation cases shown in Figure 2. The image preparation sequence of background masking, Gaussian filtering, and contrast enhancement (Figure 2E) produced the most accurate warps based on average landmark displacement and percentage of misregistered landmarks. A misregistered landmark was defined as a landmark that was off by more than one slice in the z-dimension and/or was observed to be farther than the greatest in-plane (within the x-y plane of the image) nearest-neighbor distance (i.e. 1.41 pixel lengths). Hence, a misregistered landmark was off by more than the greatest nearest-neighbor distance in 3D (1.73 pixel lengths). These criteria were used to account for both observer error and in-plane click error (the error related to using a computer mouse input device to make selections). The dashed line in Figure 4 indicates the average in-plane click error for the observer. We point out that there is little difference between the landmark results for the filtered and unfiltered images. However, direct observation of the warped and target images in an animated image sequence revealed several anatomical features (i.e. vasculature) that were poorly registered in the unfiltered images, particularly in the distal regions of the lung. We note that these features were by chance not selected as landmarks, so their misregistration is not reflected in Figure 4. Nevertheless, filtering was employed in the image processing procedure.

The accuracy of the registrations on the fully processed images (Figure 2E) was evaluated by comparing the position of all landmarks in the deformed images with respect to the target image in the three control rats. The average TRE of all landmarks was 1.31±0.54 pixels (mean ± SD), lower than the maximum nearest-neighbor distance of 1.73 pixels. Of the 67 total landmarks, 12 were found to be misregistered by at least two of the observers (TRE = 2.11±0.59 pixels), only one of which was found to be misregistered by all three observers (TRE = 2.76 pixels). The average registration errors measured by the three observers were: 1.26±0.94, 1.10±0.69, and 1.57±0.95 pixels.

**Figure 5:**
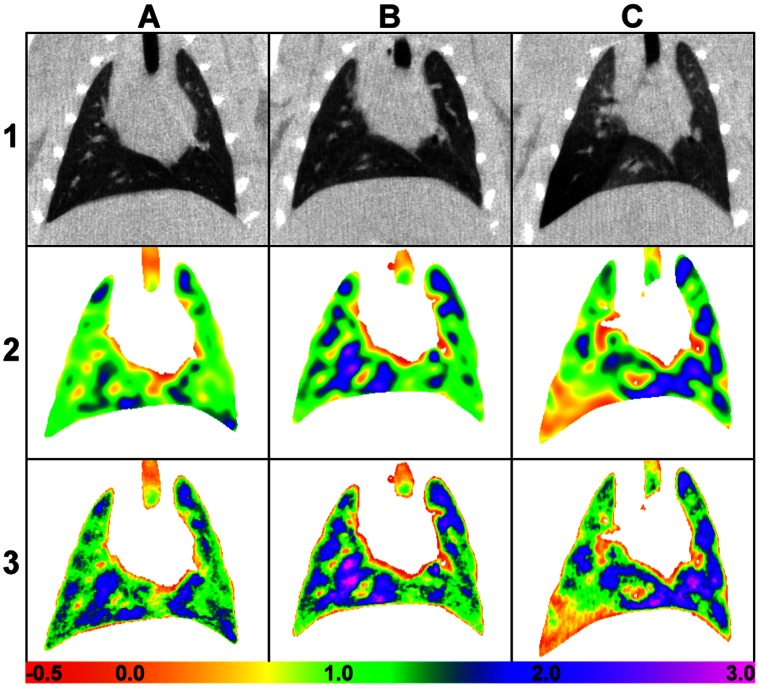
Sample registration results. Column A: control. Column B: full-lung dosed. Column C: single-lobe dosed. Row 1: coronal images, unprocessed. Row 2: Jacobian maps, between the lowest and highest inflation levels. Row 3: volume maps. The color scale represents the volume change (×10^−6^ mL) in each voxel between 0 cmH_2_O and 8 cm H_2_O.

Figure 5 shows an example of results from a representative rat from the control (column A), full-lung dosed (column B), and single-lobe dosed (column C) groups. Row 1 shows a representative coronal slice of the original target images at FRC, row 2 shows the registration Jacobian, and row 3 is the inhaled volume maps. The maps generally show heterogeneity in the air volume distribution patterns, which tends to increase in the diseased rats. The coefficient of variation (CoV), a general measure of heterogeneity, was used to compare overall volume map heterogeneity. The average CoV for the control group was 0.456±0.024, for the full-lung dose group was 0.486±0.048, and for the single-lung dose group was 0.613±0.147.

**Figure 6:**
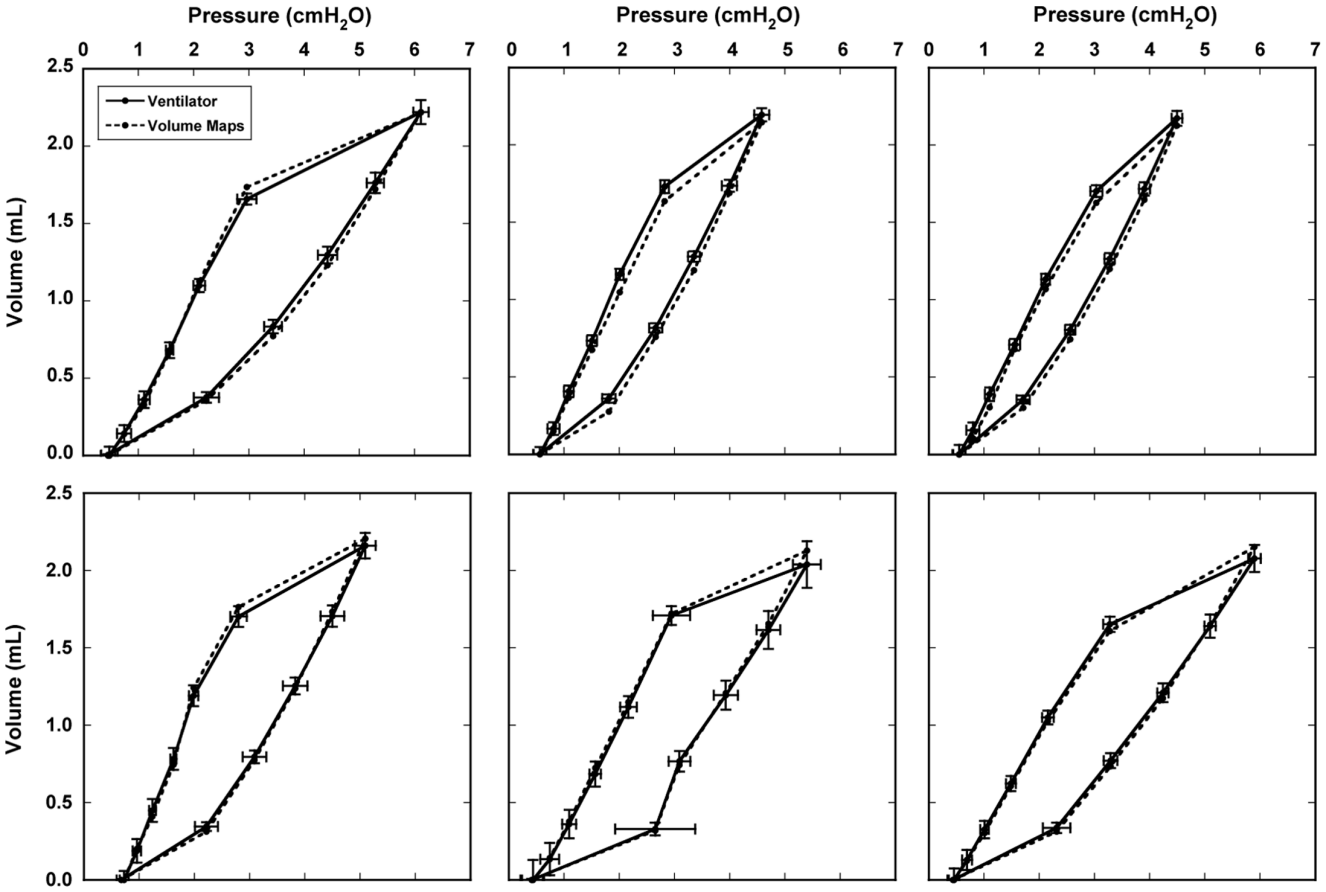
Comparison of the average ventilator-measured volume and the total volume from the volume maps for six untreated rats, plotted as a function of tracheal pressure. Error bars represent the standard deviation of the mean ventilator volume and tracheal pressure over the entire imaging experiment.

We compared the volume change from the maps at each imaging time point during the inhalation phase of the ventilation cycle to the average volume increase as measured by pneumotachographs on the inhalation and exhalation lines of the ventilator. Figure 6 shows the measured PV curve from the ventilator, as well as the PV curve computed from Equation 7, from the six additional undosed rats. The latter is the sum of the flow into each voxel that belongs to the mask obtained in Section 2.5. Error bars represent the standard deviation of the mean volume and tracheal pressure measured by the ventilator over 300–400 breathing cycles during imaging. The close agreement between the measured and calculated volume measurements is a global (whole lung) indicator of the correctness of the volume maps.

**Figure 7:**
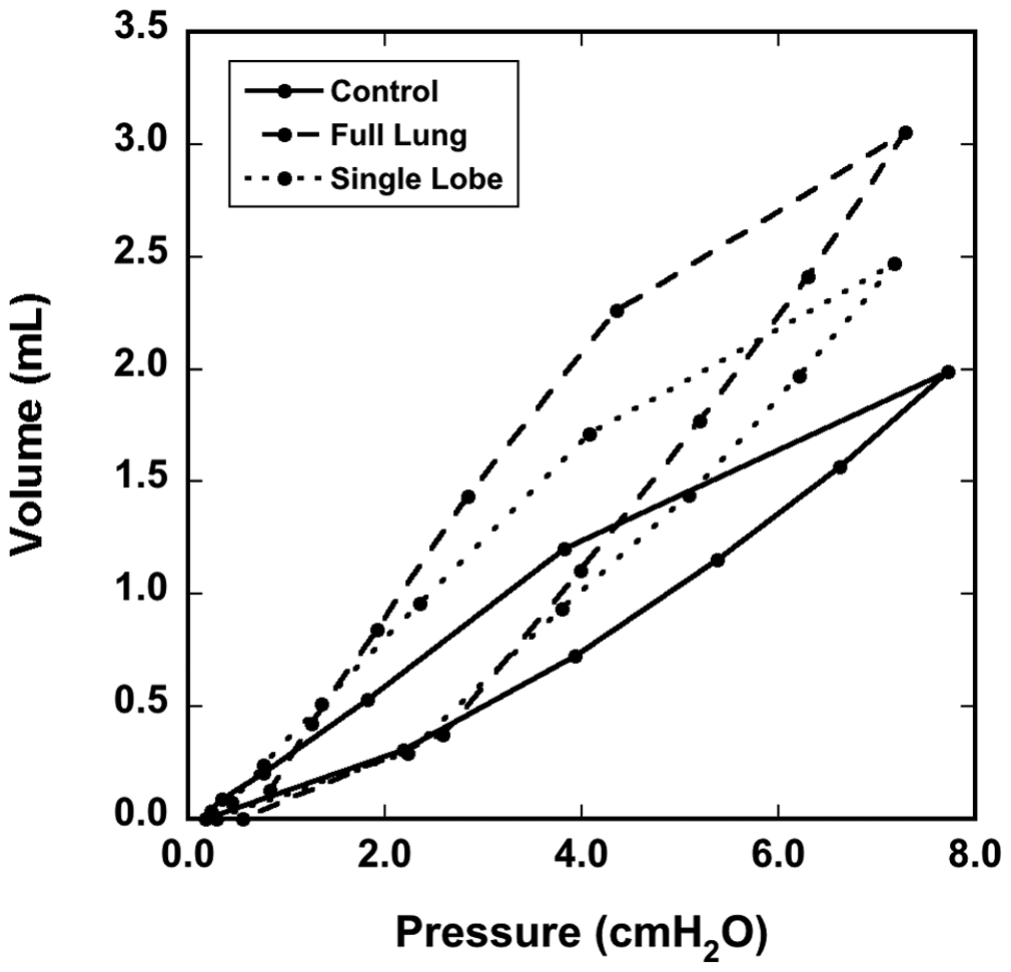
Representative pressure-volume curves made from the total volume from the volume maps and average tracheal pressure. Data are from three different rats, one from each dose group.

Figure 7 shows representative hysteresis loops of a rat in each dose group made from the volume maps and average tracheal pressures. The changes in the overall slope are indicative of changes in global lung compliance (compliance is the slope of the PV curve) due to disease. The average compliance for the control group as measured by the volume maps was 0.322±0.053 mL/cmH_2_O, for the full-lung dose group was 0.430±0.046 mL/cmH_2_O, and for the part-lung dose group was 0.367±0.007 mL/cmH_2_O. For comparison, the average compliance for the control group as measured by the ventilator was 0.325±0.046 mL/cmH_2_O, for the full-lung dose group was 0.424±0.028 mL/cmH_2_O, and for the part-lung dose group was 0.373±0.012 mL/cmH_2_O. As expected, compliance is highest in the rat that received the full-lung dose and is an intermediate value in the rat that received a partial-lung dose. We note that, despite the apparently severe disease in the dosed region of the partial-lung dosed rat, the remainder of the lung was undosed, and therefore the overall effect on the PV curve was a moderate change in compliance.

## Discussion

For the first time, we show volume maps calculated from dynamic CT images of a live, ventilated rat. Figure 6 demonstrates that the volume map calculation captures the complex nature of the lung elasticity and hysteresis, as the PV curve of the measured and calculated volumes agree. Figure 7 shows that the method is sensitive to both global and local alterations in lung mechanics caused by disease. Our ongoing work will exploit methods discussed herein to provide physiologically accurate boundary conditions in transient computational fluid dynamics models. For the models to be reliable, it is first and foremost important that the image registration faithfully represents the actual tissue displacement. To facilitate accurate warping of 4D CT rat lung images, we tested the effects of different image preparation approaches. Furthermore, we selected the two most extreme images (end inspiration and end expiration) from 4D data sets in order to provide the most stringent test for the warping procedure. Based on the landmark locations determined by the three observers, we conclude that overall the warp is generally accurate, and on average is within 1.73 pixel lengths – the maximum distance of nearest-neighbor voxels in 3D. The resulting vector fields generated by the warp were then used to calculate the Jacobian and local air volume distribution (Figure 5).

In warped images, the largest registration errors were typically observed in three regions: 1) near lobar boundaries where spatial variation of motion may be discontinuous [43] (particularly at the interface of the left and accessory lobes), 2) around the heart/diaphragm interface where motional blurring is maximum, and 3) in the conducting airways. It may be possible to address the first region with lobar segmentation and individual warping of each lobe. However, the rat lobar boundaries are typically not distinctly defined in the images, and they would have to be segmented exactly in both the target and source images to avoid compounding the registration errors. In an attempt to mitigate the second region, we developed an automated scheme to delineate the diaphragm to help the warping software better discern the true motion of the caudal regions of the lung. This approach helped somewhat in the immediate vicinity of the heart/diaphragm interface but unfortunately introduced warping artifacts in other regions, so it was abandoned. We speculate that a more sophisticated approach in which the heart was also outlined may prove beneficial; however, the lack of image contrast between the heart and surrounding tissue complicates this. In the third region, our images show that the cross-sectional area of the conducting airways expanded by 2x or more from end expiration to end inspiration (∼0 cmH_2_O to ∼8 cmH_2_O) [33], whereas vasculature flanking the airways did not expand or contract measurably. However, the warps only partially captured the expansion of most of the airways. We have not yet addressed this problem, but we speculate that airway segmentation may improve results by better defining the airways or by allowing for separate warping of the airways.

Our volume maps were qualitatively similar in appearance to those generated in the healthy rabbit using dual-energy synchrotron radiation [44] and in the healthy rat using hyperpolarized ^3^He [45] – all of which show some level of heterogeneity. It has also been shown that healthy human lungs also have ventilation defects [46]. However, severe ventilation heterogeneities are well documented in diseased lungs. We quantified the heterogeneity on a global (i.e. whole lung) level using the CoV, but this does not provide size or spatial information of regional variations or ventilation defects. In some instances the diseased regions are easily discernable in the CT images. For example, in Figure 5 panel C1 clearly shows a region of lower tissue density (indicated by lower signal intensity) in the left lobe where the elastase dose was delivered. However, the CT image does not provide any information about how well that region is ventilated. The volume map of the single-lobe dosed rat (panel C3 of Figure 5) shows comparatively little increase in air volume in the corresponding region of the left lobe. This is consistent with air trapping that can result from a severe emphysematous disease state. Indeed, the 4D CT images of this rat showed that the main airway leading to the distal portion of the left lung was completely collapsed through much of the breathing cycle, confirming the lack of change of air volume depicted in the map. However, in more subtle cases, disease presence is not always obvious, such as in the full-lung dosed rat (Column B of Figure 5). Yet our results suggest that the increased heterogeneity in the volume maps may be indicative of elastase-induced disease; further studies should be performed to verify this. Others have correlated lung motion heterogeneity with histology in a bleomycin disease model [18]. Although histology is useful for documenting physical and/or biological changes in the lung tissue that may indicate disease, it does not necessarily reveal alterations to ventilation patterns or lung motion.

Our computation of volumetric strain differs from that previously described [40], [47]–[50] in that the result is piecewise linear and the full deformation gradient is computed as opposed to simply the Jacobian. That is, we determine the deformation gradient first with a finite element discretization of the voxel and then find its determinant. In this way, we can determine the full 2nd order deformation and strain tensors that will be necessary to tease apart the change in volume (volumetric stress) from the change in shape (deviatoric stress). This will allow our future model to not only be thermodynamically correct but also to facilitate the connection to alveolar micromechanics.

There are several limitations to this work. 1) The level of anesthesia of the ventilated rats is high. We have learned through experience that rats must be deeply anesthetized to assure total compliance with the ventilator. Although the depth of anesthesia may affect breathing mechanics (and resulting CFD modeling), we are unaware of published evidence indicating such in ventilated rats. 2) We are able to collect dynamic images at a limited number of time points throughout the breathing cycle. Additional images would help define the PV hysteresis more precisely. This would be at the expense of longer imaging time, and ventilator-induced lung injury is a concern in animals ventilated for extended durations. However, others have observed minimal lung damage in rats ventilated for durations used in this study [51]–[53]. 3) We are currently unable to spatially validate the volume change maps. As a global (whole lung) confirmation of the maps, we compared inhaled gas volumes as measured by pneumotachographs with the total volume of the maps and found good agreement (see Figure 6). This provides a limited validation of our volume maps; however, it provides no spatial information. A potentially useful validation tool was recently demonstrated by [54] that directly measures local ventilation. They delivered 40 nm aerosolized fluorescent particles to rats then mapped their distribution using a cryomicrotome. By comparing the spatial distribution of inhaled nanoparticles to maps of volume change, a direct correlation between particle density and the air volume may be feasible. 4) Histology was not performed. Lungs were casted after imaging in order to provide detailed and anatomically correct airway architecture for CFD models [5] to a generation of branching that is not possible to obtain by imaging the lungs *in situ* due to lack of contrast in the deep lung. Although histology is useful to validate the severity of disease in the dosed rats, for our work this is secondary in importance to obtaining the airway geometry. 5) We measured tracheal pressure during ventilation. Measurement of pleural pressure, or esophageal pressure as a surrogate [55], gives a more precise calculation of lung compliance. 6) The radiation dose from imaging is high. Because of this, these imaging experiments are not intended to be repeated in the same animal.

Our application of this work to transient CFD models is ongoing. Local volume changes with pressure measurements determine compliance, an important model parameter. We also use the volume change maps, along with detailed airway structure obtained from *in situ* cast images, to define airflow patterns. This may prove important in predicting particulate deposition and clearance in exposure simulations.
